# Are Symbiotic Methanotrophs Key Microbes for N Acquisition in Paddy Rice Root?

**DOI:** 10.1264/jsme2.ME15180

**Published:** 2016-03-10

**Authors:** Kiwamu Minamisawa, Haruko Imaizumi-Anraku, Zhihua Bao, Ryo Shinoda, Takashi Okubo, Seishi Ikeda

**Affiliations:** 1 Graduate School of Life Sciences, Tohoku UniversityKatahira, Aoba-ku, Sendai, MiyagiJapan; 2 Department of Plant Sciences, National Institute of Agrobiological SciencesTsukubaJapan; 3 College of Environmental and Resource Science, Inner Mongolia UniversityWest University Blvd., Hohhot, Inner Mongolia Autonomous RegionPR China; 4 Memuro Research Station, National Agricultural Research Center for Hokkaido RegionShinsei, Memuro-cho, Kasaigun, HokkaidoJapan

**Keywords:** methane oxidation, nitrogen fixation, symbiosis, paddy rice, nitrogen fertilizer

## Abstract

The relationships between biogeochemical processes and microbial functions in rice (*Oryza sativa*) paddies have been the focus of a large number of studies. A mechanistic understanding of methane–nitrogen (CH_4_–N) cycle interactions is a key unresolved issue in research on rice paddies. This minireview is an opinion paper for highlighting the mechanisms underlying the interactions between biogeochemical processes and plant-associated microbes based on recent metagenomic, metaproteomic, and isotope analyses. A rice symbiotic gene, relevant to rhizobial nodulation and mycorrhization in plants, likely accommodates diazotrophic methanotrophs or the associated bacterial community in root tissues under low-N fertilizer management, which may permit rice plants to acquire N via N_2_ fixation. The amount of N fixed in rice roots was previously estimated to be approximately 12% of plant N based on measurements of ^15^N natural abundance in a paddy field experiment. Community analyses also indicate that methanotroph populations in rice roots are susceptible to environmental conditions such as the microclimate of rice paddies. Therefore, CH_4_ oxidation by methanotrophs is a driving force in shaping bacterial communities in rice roots grown in CH_4_-rich environments. Based on these findings, we propose a hypothesis with unanswered questions to describe the interplay between rice plants, root microbiomes, and their biogeochemical functions (CH_4_ oxidation and N_2_ fixation).

Flooded fields such as rice (*Oryza sativa*) paddies are a major source of atmospheric CH_4_, a powerful greenhouse gas, via biogeochemical processes that are mediated by soil and plant microbial communities (31, 45, 61). Microbial processes relevant to the CH_4_ cycle are not fully understood even by omic–driven and culturing approaches (43). The ecosystem of rice paddies has been regarded as an ideal model system for studies on the fundamental aspects of microbial ecology (29, 36, 41). The rhizosphere is regarded as a hot spot for the transformation of a number of inorganic and organic substances, including C1 compounds such as methane (CH_4_), by means of redox reactions (29, 36, 41). CH_4_ produced from anoxic soils by methanogenic archaea is transported from the roots to the leaf sheaths via the aerenchyma of the rice plant (44). On the other hand, rice roots in paddies and rhizosphere soil grow under partially oxic conditions, allowing the growth of aerobic methanotrophic bacteria that utilize CH_4_ and methanol as their carbon and energy sources (17). Up to 90% of CH_4_ is consumed by aerobic methanotrophs in the rice root (21, 38, 61).

Nitrogen (N) is one of the most important nutrients for plant growth (30). Although modern agriculture depends heavily on an adequate supply of N to sustain high crop yields, this is accompanied by well-documented high energy costs and environmental damage (30). Thus, reduced fertilizer usage is one of the objectives of field management to promote sustainable agriculture. Bodelier *et al.* (6) found that ammonium-based fertilizers stimulated CH_4_ oxidation in the soil around rice roots and reduced the emission of CH_4_. Other researchers also reported that N fertilization levels affect CH_4_ emission from rice fields; however, the details of this topic are being debated (3, 53, 62). A mechanistic understanding of CH_4_–N cycle interactions is a key unresolved issue in biogeochemical research on rice paddies and natural wetlands (7–9, 15).

Recent multi-omic approaches have provided insights into the functional dynamics of CH_4_–N cycles in freshwater lakes (12) and permafrost ecosystems (22, 32). However, few studies have examined CH_4_–N cycle interactions in rice paddies and wetland soils (*e.g.*, 9). Plant-associated bacteria often occupy endophytic niches in the plant roots and shoots (25). Until recently, few such analyses, including those based on metagenomics and metaproteomics, had been applied to endophytes due to the technical difficulties associated with preparing metagenomic microbial DNA and proteins without serious contamination by plant materials. A technique to enrich bacterial cells from plant tissues has been developed (24) and was shown to be useful for analyses of the microbiomes associated with rice roots, including those of bacterial endophytes and epiphytes (25, 27–29, 45).

A metagenomic study (28) indicated that low-N fertilization management strongly affected the biogeochemical processes in rice roots in a paddy field ecosystem, in which three key players (including methanotrophic *Methylosinus* sp.) were identified in the bacteria associated with rice roots under low levels of N fertilizer application (28). Subsequent research (4, 5) suggested interplay between a plant symbiosis gene, CH_4_ oxidation, and N_2_ fixation in rice roots in paddy fields. Since this interplay occurred exclusively under low-N fertilization management, mediated through the plant symbiosis gene, these processes are likely to be similar to symbiotic N_2_ fixation between rhizobia and legumes. Based on these studies, we propose a hypothesis for unanswered questions on the interplay between rice plants, root microbiomes, and their biogeochemical functions.

## Bacterial community shifts

**Fig. 1:**
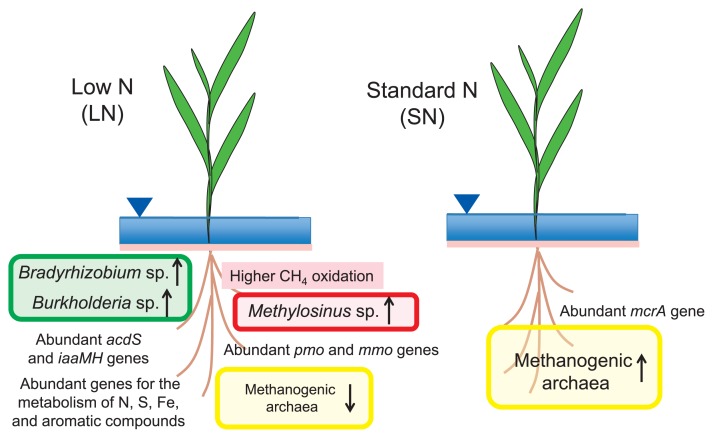
Schematic representation of differences in bacterial communities in and around paddy rice roots grown under low-N (LN) and standard-N (SN) fertilization conditions (28).

The level of N fertilizer is a crucial factor that shapes the bacterial community in field-grown plants (25, 26). Ikeda *et al.* (28) analyzed the bacterial communities associated with rice plants (cv. ‘Nipponbare’) in paddy fields with low and standard levels of N fertilizer application (LN and SN at 0 and 30 kg N ha^−1^, respectively, with N supplied as urea). Culture-independent community analyses based on 16S rRNA gene sequences indicated that the root microbiome responded strongly to the level of N fertilization (Fig. 1). The relative abundances of three operational taxonomic units (OTUs) in the genera *Methylosinus*, *Bradyrhizobium*, and *Burkholderia* were significantly higher in the root microbiome of the LN field than in that of the SN field based on statistical metagenome analyses (28). In contrast, the abundance of methanogenic archaea showed the opposite pattern (28). Proteobacterial methanotrophs were classified into two groups: the family *Methylocystaceae* (type II methanotrophs) belongs to the class *Alphaproteobacteria*, and the family *Methylococcaceae* (type I methanotrophs) belongs to the class *Gammaproteobacteria* (17). The methanotrophs associated with rice roots were exclusively classified as type II methanotrophs (28).

The functional genes for CH_4_ oxidation (*pmo* and *mmo*) and plant association (*acdS*) were significantly more abundant in the LN root microbiome. In addition, functional genes for the metabolism of N, S, Fe, and aromatic compounds were more abundant in the LN root microbiome (28) (Fig. 1). Sessitsch *et al.* (52) performed a metagenomic analysis of the bacterial endophyte community from surface-sterilized rice roots, and found many genes associated with an endophytic lifestyle, such as plant polymer–degrading enzymes and the detoxification of reactive oxygen species. However, our findings demonstrated the strong influence of geochemical and nutritional environments, and this may be because of the enriched bacterial cells in rice roots including epiphytes and endophytes under low-N fertilization (24).

A metagenome analysis generally shows the relative abundance of microbial species and their potential functional genes (9, 12). However, quantitative PCR for *pmoA* (encoding methane monooxygenase) and *mcrA* (encoding methyl coenzyme-M reductase) and a ^13^C-labeled CH_4_ experiment provided evidence of more active CH_4_ oxidation in the rice roots of the LN field than in those of the SN field (28). These findings suggest that low-N fertilizer management is an important factor shaping the microbial community structure, which contains key plant-associated microbes that are involved in biogeochemical processes in paddy rice ecosystems.

## Plant genes for microbial symbioses

**Fig. 2:**
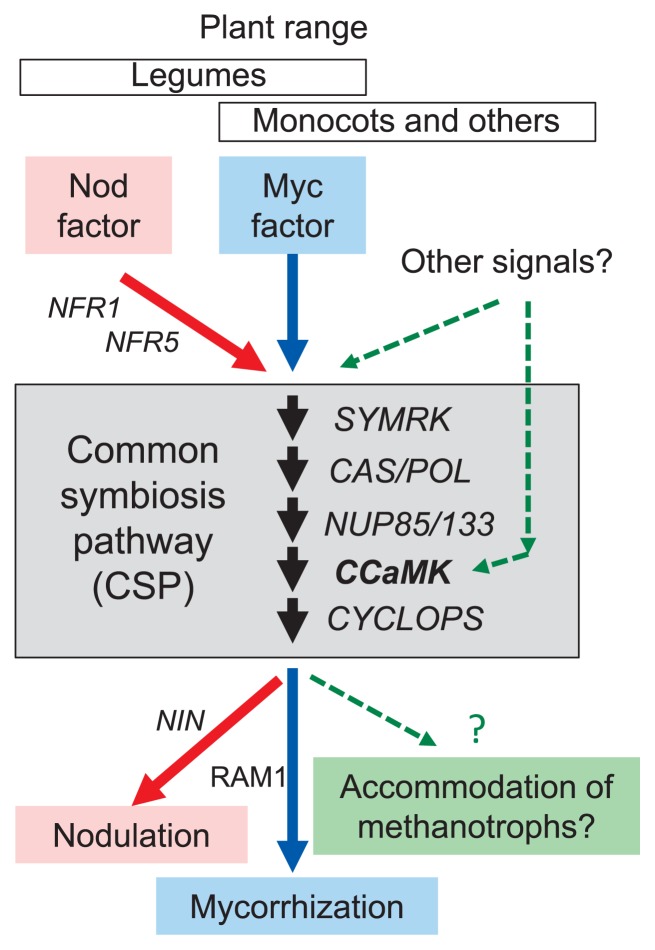
The common symbiosis pathway (CSP) in plants. The signal-transduction pathway of symbiosis with rhizobia and mycorrhizae is based on recent research (37, 47, 54–56, 59). *CCaMK* (which encodes a Ca^2+^/calmodulin–dependent protein kinase) is a key player for microbial symbiosis in the CSP (37, 47, 54–56, 59). The green dotted line shows the presumed pathways described in this minireview (see the text for details).

Leguminous plants have evolved mutual symbioses with rhizobia and mycorrhizae (37, 47, 54, 56, 59). The genetic requirements for rhizobial and mycorrhizal interactions in plants overlap in a common symbiosis pathway (CSP), which leads to successful symbioses (Fig. 2; 37, 47, 54, 59). Non-leguminous plants have mutualistic symbiotic relationships with arbuscular mycorrhizal fungi through the CSP (37, 42, 55). Ca^2+^/calmodulin–dependent protein kinase (encoded by *CCaMK*) has been identified as a key component of the CSP, which is required for rhizobial and mycorrhizal endosymbioses to take up N and phosphorus, respectively (37, 47, 54–56, 59). The CCaMK protein decodes Ca^2+^ spikes triggered by microbial oligosaccharide signals (Nod or Myc factors), and phosphorylates CYCLOPS, which activates downstream symbiotic gene expression (37, 47, 54, 56, 59). Orthologs of CSP genes, including *CCaMK* (*DMI3*), *CYCLOPS* (*IPD3*), and *POLLUX* (*DMI1*), are well conserved in non-leguminous monocot plants (47, 54, 55) and also in liverworts and hornworts (60). The RiceXPro expression database (51) indicates that the *Oryza sativa CCaMK* (*OsCCaMK*; Os05g0489900) genes were constitutively expressed in the roots of field-grown rice under natural field conditions at the vegetative and reproductive stages (27). In addition, a mutant line with a defective *Oryza sativa CCaMK* (*OsCCaMK*) showed greatly reduced mycorrhization in rice roots (2).

In field experiments (27), rice roots of the *OsCCaMK* mutant described above had a lower relative abundance of members of the order *Rhizobiales*, which include rhizobia, CH_4_-oxidizing bacteria (type II methanotrophs), and N_2_-fixing bacteria. These findings raised the question of whether the *OsCCaMK* genotype affects the composition of root-associated bacteria that are related to the C and N cycles in paddy fields. However, little is known about the interactions between *OsCCaMK* and the bacterial community associated with rice roots.

## Methane flux and methanotrophs

**Fig. 3:**
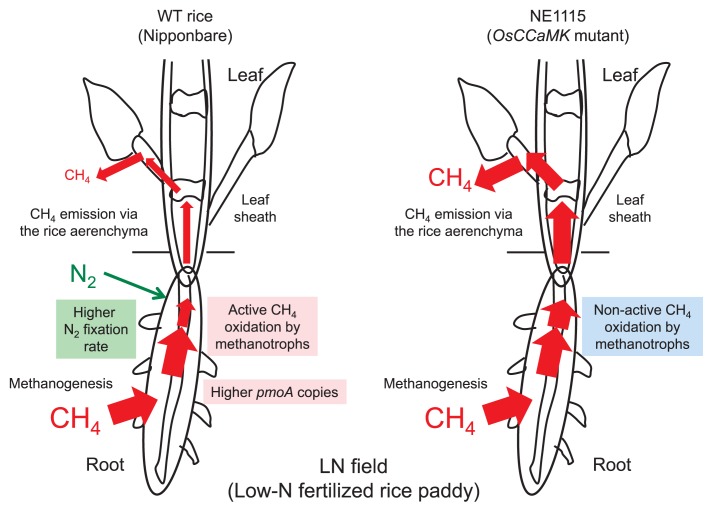
Schematic representation of differential CH_4_ fluxes from WT rice and NE1115 (with defective *OsCCaMK*) grown in the paddy field under the low-N fertilization condition (5).

Given the above background, studies were conducted to investigate whether *OsCCaMK* regulates microbial CH_4_ oxidation and N_2_ fixation in the roots of rice plants in paddy fields with LN and SN fertilization histories (0 and 30 kg N ha^−1^, respectively). The effects of the *OsCCaMK* mutant on the soil CH_4_ flux were compared with those of the wild-type (WT) in the LN and SN paddy fields during a 2-year study using cv. ‘Nipponbare’. The *OsCCaMK* mutant grew less than the WT, particularly in the LN field. The CH_4_ flux from the *OsCCaMK* mutant in the LN field was significantly and consistently higher than that in the WT field throughout the growing season (Fig. 3; 5). On the other hand, no significant difference was observed in the CH_4_ flux between the *OsCCaMK* mutant and WT in the SN field (5).

Since the CH_4_ cycle in a rice paddy is complex (17, 29, 36, 41, 44), careful evaluations were carried out to determine why the CH_4_ flux of the *OsCCaMK* mutant was significantly higher than that of the WT in the LN field. The tiller numbers and aerenchyma morphology were similar between the *OsCCaMK* mutant and WT in the LN field. The dissolved soil CH_4_ concentration and *mcrA* copy number also showed no significant difference between the WT and *OsCCaMK* mutant in the LN field. These results suggest that microbial factors relevant to CH_4_ oxidation, rather than plant morphological factors or archaeal CH_4_ production, were the primary explanation for the enhanced CH_4_ flux by the *OsCCaMK* deficiency in the *OsCCaMK* mutant. CH_4_-oxidizing activity and the *pmoA* copy number were higher in the roots of the WT than in the roots of the *OsCCaMK* mutant. These findings strongly suggest that the greater CH_4_ flux in the *OsCCaMK* mutant was attributable to a decrease in CH_4_ oxidation rather than to an increase in CH_4_ production (Fig. 3; 5).

## Contribution of nitrogen fixation

In order to estimate the extent of N_2_ fixation, natural N isotope abundance (δ^15^N) was determined in the shoots of rice plants grown in the LN paddy field. The natural abundance of ^15^N in the WT shoots (3.05‰) was significantly lower than that in the *OsCCaMK* mutant shoots (3.45‰), suggesting higher N_2_ fixation in the WT due to dilution with atmospheric N_2_ (^15^N natural abundance, 0.00‰; 5). We made two assumptions: (i) that all N in *OsCCaMK* mutant shoots was derived from soil N and (ii) that the abundance of ^15^N in the WT shoots (3.05‰) was diluted more than that of ^15^N in *OsCCaMK* mutant shoots (3.45‰) due to mixing with atmospheric N (^15^N abundance, 0.00‰). On assumption (ii), ^15^N isotope discrimination during N_2_ fixation was ignored because it is almost zero in symbiotic systems (58). On this basis, we estimated the contribution (%) of biological nitrogen fixation to total N of the WT rice plant (BNF, %) as follows:

$${Ndf}_{\text{a}}\times 0.00+{Ndf}_{\text{s}}\times 3.45=({Ndf}_{\text{a}}+{Ndf}_{\text{s}})\times 3.05$$

In this equation, *Ndf*_s_ and *Ndf*_a_ represent the amounts of N in WT shoots derived from soil N and atmospheric N, respectively. As a result, it is possible to calculate biological nitrogen fixation (BNF) (%) as follows:

$$\text{BNF }(\%)={Ndf}_{\text{a}}/({Ndf}_{\text{a}}+{Ndf}_{\text{s}})\times 100=11.6\%$$

This estimate suggests that N_2_ fixation in WT rice was 11.6% higher than that in the *OsCCaMK* mutant NE1115 in a paddy rice field experiment (Fig. 3).

Therefore, CH_4_ oxidation and N_2_ fixation were simultaneously activated in the roots of WT rice in the LN field, and both processes are likely controlled by *OsCCaMK*. In order to confirm the interplay between these processes, further experiments will be required to clarify the effects of N levels and the *OsCCaMK* mutation on the activity of CH_4_-dependent N_2_ fixation.

## Which organisms fix nitrogen in rice roots?

The next important question is to determine which microorganisms in rice roots are responsible for CH_4_ oxidation and N_2_ fixation in the LN field. Two possibilities have been suggested: (i) methanotrophs mediate CH_4_ oxidation and N_2_ fixation, and (ii) methanotrophs mediate CH_4_ oxidation only, whereas other diazotrophs fix N_2_ by using intermediate substrates such as methanol during CH_4_ oxidation. Since bacterial proteins were easily prepared from the bacterial cells purified from rice roots (24), metaproteomics was a suitable approach to answer this question.

### Methanotrophs

It is well known that type II methanotrophs fix atmospheric N_2_ (17). A metaproteomic analysis of root-associated bacteria from field-grown rice roots revealed that dinitrogenase reductase (NifH) and the alpha subunit (NifD) and beta subunit (NifK) of dinitrogenase were mainly derived from type II methanotrophic bacteria of the family *Methylocystaceae*, which includes *Methylosinus* spp. Minor nitrogenase proteins from *Methylocella*, *Bradyrhizobium*, *Rhodopseudomonas*, and *Anaeromyxobacter* species were also detected. Methane monooxygenase proteins (PmoCBA and MmoXYZCBG) were frequently detected in the same bacterial group as the *Methylocystaceae* (4). These findings suggest that *Methylocystaceae* members mediate CH_4_ oxidation and N_2_ fixation.

**Fig. 4:**
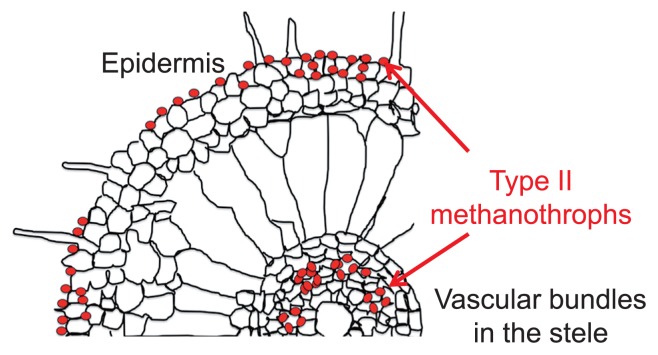
Schematic presentation of CARD-FISH microscopic observations of type II methanotrophs in rice root tissues (4). Red indicates the colonization positions of type II methanotrophs.

The localization of type II methanotrophic bacteria was subsequently examined in the tissues of field-grown rice by using catalyzed reporter deposition–fluorescence *in situ* hybridization (CARD-FISH). Type II methanotrophs were localized around the epidermal cells and vascular cylinders in the root tissues of field-grown rice plants (Fig. 4), indicating that they have endophytic and epiphytic lifestyles in rice roots. The findings of the metaproteomic and CARD-FISH analyses suggest that CH_4_ oxidation and N_2_ fixation are mainly performed by type II methanotrophs, including *Methylosinus* spp., which inhabit the vascular bundles and epidermal cells of rice roots (4).

### Consortia including methanotrophs

A metaproteomic analysis is a powerful approach for identifying microbes and their biogeochemical pathways. Knief *et al.* (35) applied this approach to epiphytes on rice plants and found that many proteins were involved in the CH_4_ cycle and N_2_ fixation in the rhizosphere. Although CH_4_ oxidation and N_2_ fixation were both mainly mediated through type II methanotrophs in rice roots (4), it is possible that *Bradyrhizobium* spp. are N_2_-fixing bacteria in rice roots. There are several reasons for this. Bradyrhizobial NifD and MDH (methanol dehydrogenase) proteins were detected as minor components in the LN root microbiome (4). Furthermore, our research group isolated three major bacteria (*Methylosinus*, *Bradyrhizobium*, and *Burkholderia*), the abundances of which increased in the LN root microbiome (28) (Bao *et al.*, unpublished results; Shinoda *et al.*, unpublished results). The isolation strategies for these organisms were based on their respective traits using the 16S rRNA sequence as a marker gene (1, 48).Genomic and phenotypic examinations revealed that the *Bradyrhizobium* and *Methylosinus* isolates were both N_2_-fixing bacteria. Additionally, methanotrophs often secrete methanol and develop consortia with methylotrophs, which has long been known as a contamination issue in efforts to obtain pure cultures of methanotrophs (17). Ho *et al.* (19) found a significant increase in CH_4_ oxidation with enhanced heterotroph richness, suggesting that complex, but crucial interactions lead to the stimulation of CH_4_-oxidizing activity.

The *xoxF* genes encoding a methanol dehydrogenase (MDH), which depends on certain rare earth elements (REE-binding XoxF-MDH), were recently found to be widely distributed in bacteria in natural environments, including bradyrhizobia, rather than conventional *maxFI* genes that encode Ca-binding MaxFI-MDH (14, 34). Since the soil concentrations of lanthanum (La) and cerium (Ce) are sufficient to support XoxF-MDH activity (34, 57), it is possible that methanotrophs carrying XoxF-MDH form consortia that support CH_4_-dependent N_2_ fixation in the presence of the REEs. In a soil experiment with CH_4_ and ^15^N_2_ enrichment, ^15^N-labeled *nifH* genes were detected in members of *Methylosinus* and *Rhizobiales* (10). Similar consortia that engaged in cobalamin transfer were detected in a CH_4_-enriched culture (23).

Taken together, it is possible that a consortium composed of *Methylosinus* and *Bradyrhizobium* (or other methylotrophs) behaves as a methanotrophic diazotroph that is mediated by the production of methanol. Such a consortium may overcome the negative effects of energy competition that occur in methanotrophic diazotrophs (7–9).

## Environmental responses of methanotrophs

Populations of type II methanotrophs in rice roots respond well to the environments of rice paddies, even under standard N fertilization. Root methanotrophs were found to increase along with CH_4_ emission levels from the rice panicle initiation to ripening stages (45). In addition, abundances in the ripening stage were increased by soil warming (2°C higher than the natural temperature) and decreased by CO_2_ enrichment (200 ppm higher than the ambient CO_2_ concentration) (45). Members of bradyrhizobial cluster I (phylogenetically close to photosynthetic N_2_-fixing bradyrhizobia) fluctuated in a similar manner to type II methanotrophs (45), supporting the above idea of diazotrophic consortia. Decreases in the abundance of methanotrophs and a decreased copy number of *pmoA* have been reported across different varieties of rice under CO_2_ enrichment (46). These findings showed that type II methanotrophs in rice roots are sensitive to changes in environmental characteristics such as temperature and CO_2_ concentrations, suggesting that rice plants regulate methanotrophs via changes in the physiological status of rice plants. Therefore, CH_4_ oxidation mediated by methanotrophs is a driving force in shaping bacterial communities in rice roots grown in CH_4_-rich environments.

## A hypothesis for the methane–nitrogen cycle in rice roots

**Fig. 5:**
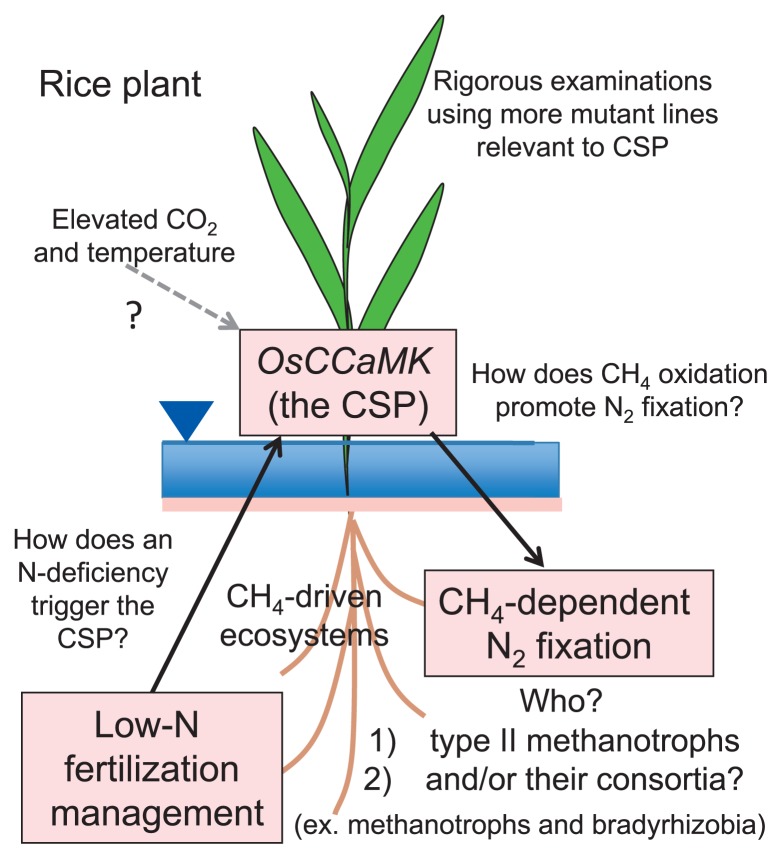
Hypothetical relationships between N fertilizer management, the CSP, and CH_4_-dependent N_2_ fixation (4, 5, 28).

Based on the research summary provided in this minireview, we will summarize how these findings on rice root microbiomes describe the responses that occur under low-N fertilization management, and propose a hypothesis plus unanswered questions to describe the interplay among rice plants, their root microbiomes and functions (CH_4_ oxidation and N_2_ fixation), and N fertilizer management (Fig. 5).

Research results have revealed that low-N fertilization management and the rice *OsCCaMK* genes strongly affect the biogeochemical processes related to rice roots in a paddy field ecosystem, including CH_4_-dependent N_2_ fixation. Rice plants in the LN field appeared to recognize the history of low-N fertilization management by unknown mechanisms, and, thus, identifying these mechanisms is important because of their potential to promote sustainable agricultural practices with lower fertilization or possibly even without fertilization. Since rice line NE1115, an *OsCCaMK* mutant, showed markedly decreased levels of type II methanotrophs and decreased CH_4_ oxidation and N_2_ fixation in the LN field, this gene appears to be essential to allow the development of symbioses with methanotrophs and functional CSP (Fig. 2); however, rigorous examinations using more lines with mutations that affect the CSP are needed.

There is no molecular evidence to show that the CSP is involved in the accommodation of rice to endophytic methanotrophs. Plant hormones and microbial elicitors may also be involved in these processes because they are regarded as important factors for N signaling in interactions between endophytic bacteria and plants (11). Evangelists *et al.* (13) also reported that a possible mechanism underlying the fine-tuning of root microbiomes by *CCaMK* is its role in abscisic acid (ABA) signaling and reactive oxygen species homeostasis. Similar CH_4_–N cycle interactions were found in the methanotrophs associated with submerged mosses (8, 39). These interactions may be symbiotic and mutually beneficial for both the bacteria and mosses (20, 40, 49), which may, thus, have a CSP similar to that of rice plants (60).

Symbiotic plant-microbe interactions generally exert nutrient exchanges. An adequate CH_4_ supply started at the panicle initiation stage of paddy rice plants (45); therefore, we suspect that type II methanotrophs utilize organic substrates other than CH_4_ during initial interactions with rice roots. Type II methanotrophs are known to metabolize aromatic and alicyclic compounds as well as CH_4_ via soluble methane monooxygenase (sMMO) (33). Thus, rice plants may provide these compounds to accommodate the methanotrophs in their roots before an adequate CH_4_ supply from rice paddies.

Groten *et al.* (16) recently reported that silencing *CCaMK* in *Nicotiana attenuata* did not influence root-associated microbial communities or plant growth, even under phosphorus-limited conditions. We observed a clear phenotypic difference in growth and the CH_4_ flux between WT rice and the *OsCCaMK* mutant NE1115 under low-N (LN) fertilization (5). Thus, an N deficiency in rice plants may uniquely induce signal transduction via *CCaMK* and subsequent CH_4_–N cycle interactions (Fig. 5).

Rogers and Oldroyd (50) discussed the possibility of biotechnological solutions that transfer the symbiotic association of N_2_-fixing bacteria in leguminous plants into non-leguminous cereal crops by engineering components of the CSP, including *CCaMK*. We here emphasize that rice plants, and possibly other marsh species, may already have the potential for CH_4_-dependent N_2_ fixation during their adaptation to CH_4_-enriched environments (*i.e.*, rice paddies) under N-deficient conditions. Based on research backgrounds and advances, the potential of plant-associated microbes may be maximized by new agricultural management such as symbiotic crop breeding and controlled-release N fertilizer (18). In this regard, rice varieties with a higher root biomass may be important for enhancing CH_4_ oxidation and N_2_ fixation because the cell densities of methanotrophs and/or diazotrophs in the niche of rice roots appear to be lower than that of legume nodules.
